# Racial/ethnic disparities in prevalence, treatment, and control of hypertension among US adults following application of the 2017 American College of Cardiology/American Heart Association guideline

**DOI:** 10.1016/j.pmedr.2019.100850

**Published:** 2019-03-16

**Authors:** Gulam Muhammed Al Kibria

**Affiliations:** University of Maryland School of Medicine, Baltimore, MD 21201, USA

**Keywords:** ACC/AHA, American College of Cardiology/American Heart Association, BP, Blood pressure, CI, Confidence interval, CKD, Chronic kidney disease, CVD, Cardiovascular disease, DBP, Diastolic blood pressure, JNC, Joint National Committee, NHANES, National Health and Nutrition Examination Survey, SBP, Systolic blood pressure, SE, Standard error, Hypertension, Blood pressure, Blood pressure control, Hypertension treatment, 2017 ACC/AHA, Race, Racial/ethnic disparities, NHANES, US

## Abstract

The 2017 American College of Cardiology/American Heart Association (ACC/AHA) Guideline for the Prevention, Detection, Evaluation, and Management of High Blood Pressure in Adults recommends reduced systolic/diastolic blood pressure (SBP/DBP) cutoffs to define hypertension (i.e., by changing these from ≥140/90 to ≥130/80 mmHg), including new recommendations about indications and goals of antihypertensive treatment. This study reported the differences in age-adjusted prevalence and treatment status of hypertension according to race among US adults per the 2017 ACC/AHA guideline.

The National Health and Nutrition Examination Survey 2011–16 data was analyzed. The main outcomes were age-adjusted prevalence and treatment status of hypertension among adults aged ≥20 years. After prevalence estimation, other proportions were obtained.

The analysis included 16,103 adults (mean age: 47.6 years, 51.8% women). The age-adjusted proportions of adults with hypertension (59.0%, 95% confidence interval [CI]: 57.4%–60.6%), treatment-eligible for hypertension (49.3%, 95% CI: 47.7%–50.8%), and unmet treatment goals (63.8%, 95% CI: 60.0%–67.5%) among the treated were highest among non-Hispanic blacks. A large proportion of Mexican-Americans (46.5%, 95% CI: 42.0%–51.0%) and people of other races/ethnicities (49.3%, 95% CI: 45.5%–53.0%) were not receiving treatment despite having indication. Non-Hispanic blacks also had the highest prevalence of stage 2 hypertension.

Among all races, prevalence, treatment-eligibility, and unmet treatment goals were higher among people with older age, male gender, diabetes, higher body weight, and higher cardiovascular disease risk while the majority of younger, lower/normal body weight, or non-diabetic people were untreated despite being eligible for treatment.

The prevalence, treatment-eligibility, and unmet goals were substantially higher among non-Hispanic blacks. Moreover, disparities exist in treatment where Mexican-Americans and people of ‘other races/ethnicities’ were largely untreated despite having indication.

## Highlights

• The prevalence of hypertension differed by race with the highest prevalence among non-Hispanic blacks.
• Non-Hispanic blacks also had the highest proportion of people with treatment-eligibility and unmet treatment goals.
• A large proportion of Mexican-Americans and people with ‘other races/ethnicities’ were untreated.

## 1 Introduction

Uncontrolled hypertension is the most common risk factor for cardiovascular disease (CVD) and is responsible for a large number of deaths and disabilities within the US (Forouzanfar et al., 2017; Heron, 2018). The 2017 American College of Cardiology/American Heart Association (ACC/AHA) Guideline for the Prevention, Detection, Evaluation, and Management of High Blood Pressure in Adults has changed the systolic/diastolic blood pressure (SBP/DBP) thresholds to define hypertension (Whelton et al., 2017). Previous guidelines such as the Seventh Joint National Committee Guideline (JNC 7) recommends the SBP/DBP thresholds as 140/90 mmHg; however, the new guideline recommends the cutoffs as 130/80 mmHg, 10 mmHg lower than the SBP/DBP thresholds of the previous guidelines (Chalmers et al., 1999; Chobanian et al., 2003; Whelton et al., 2017). Furthermore, the term ‘prehypertension’ has been replaced with a new term ‘elevated BP’ which recommends the SBP cutoff as 120–129 mmHg while the person has a normal DBP (i.e., <80 mmHg) (Whelton et al., 2017). The 2017 ACC/AHA guideline has also changed the recommendations about treatment initiations and treatment goals. More than 45% of US adults (i.e., aged ≥20 years) could be hypertensive as per the new guideline (Muntner et al., 2017). Other studies also support this finding (Dorans et al., 2018; Khera et al., 2018). Although this guideline has classified a substantial proportion of adults as hypertensive who would have been known as prehypertensive per the previous guidelines, it provides a greater opportunity to take prevention and treatment measures in the early stages of hypertension (Muntner et al., 2017; Whelton et al., 2017). Additionally, adoption of the new guideline could substantially reduce the cardiovascular events resulting from hypertension (Bundy et al., 2018; Whelton et al., 2017; Yano et al., 2018).

Studies from the US have consistently reported that the prevalence and likelihoods of hypertension differ by race. According to Dorans et al., the age-adjusted prevalence of hypertension was about 8.5% higher among non-Hispanic blacks compared to non-Hispanic whites in 2015–16. In addition to non-Hispanic blacks, the age-adjusted prevalence of hypertension was higher among Mexican-Americans or other races (Dorans et al., 2018). The proportion of people who would require pharmacotherapy for hypertension or who have unmet treatment goals could also be substantially higher among non-Hispanic blacks compared to other races (Muntner et al., 2017). Moreover, studies reported that Mexican-Americans could remain untreated despite having indications for treatment (Gu et al., 2017; Park et al., 2018). Differences in awareness, lifestyle, and socioeconomic characteristics could be responsible for these disparities (Gu et al., 2017; Whelton et al., 2017). Considering the clinical and public health importance of hypertension, estimates of prevalence, control, or treatment of hypertension according to characteristics such as age, gender, kidney disease, body weight, or cholesterol levels among racial groups in the US are also essential to investigate. While earlier studies found that the new guideline could change the estimates of overall hypertension burden per race in the US, how these estimates of hypertension could differ according to characteristics among different races have not been estimated yet. Furthermore, there have been limited recent studies that investigate racial disparities related to prevalence, control, and treatment of hypertension together. To understand the racial disparities related to hypertension burden, new studies are needed that quantify the prevalence, control, and treatment status of hypertensive individuals according to race with recent data per the latest guideline. To address these gaps in the literature, this study obtained the aforementioned estimates by race among US adults.

## 2 Methods

### 2.1 Data source

This study used the National Health and Nutrition Examination Survey (NHANES) data. The continuous NHANES is a cross-sectional survey. The primary aim of this biennial survey is to obtain nationally representative samples of the non-institutionalized US population. Details of the survey are available elsewhere (Zipf et al., 2013). This analysis was limited to 2011–16 survey years to report the most recent estimates. These publicly available datasets were downloaded and merged according to unique identification numbers. The Ethics Review Board of the National Center for Health Statistics approved the survey protocols (National Center for Health Statistics, 2017).

### 2.2 Study variables

Using factory-calibrated ‘Baumanometer® mercury true gravity wall model sphygmomanometers’, trained physicians measured the BP in mobile examination centers. The BP was recorded for four times in the seated position after resting for 5 min. Appropriate cuff sizes were used (Centers for Disease Control and Prevention, 2009; Zipf et al., 2013). In this study, the mean of the first 3 BP measurements was used to calculate BP levels. If an individual had an SBP of 120–129 mmHg with a normal DBP (i.e., <80 mmHg), then the BP level was classified as elevated BP. To classify an individual as stage 1 and stage 2 hypertensive, the following SBP/DBP thresholds were used, respectively (in mmHg), 130–139/80–89 and ≥140/90. Hypertension includes both stage 1 and stage 2 hypertension. Individuals who reported that they were currently taking BP lowering drugs were categorized as hypertensive regardless of BP levels (Supplemental Table 1) (Muntner et al., 2017; Whelton et al., 2017).

Participants reported their race/ethnicity, and were grouped as non-Hispanic whites, non-Hispanic blacks, Mexican-Americans, and other races/ethnicities. Due to a lower proportion of respondents from races/ethnicities other than the first 3 races, all other races (i.e., non-Hispanic Asians, other Hispanics, and other races including multi-races) were grouped as other races/ethnicities. Participants also reported their age (in years) and gender. Age was stratified into 20–44, 45–54, 55–64, and ≥65 years (Muntner et al., 2017). Borderline elevated and high cholesterol levels were defined as 200–239 and ≥240 mg/dl cholesterol levels, respectively. If the high-density lipoprotein (HDL) was <40 mg/dl for men and <50 mg/dl for women, it was categorized as low (Ostchega et al., 2018). Self-reports for diagnosis of prediabetes and diabetes were obtained. The chronic kidney disease Epidemiology (CKD-EPI) equation was used to estimate the glomerular filtration rate (GFR). A person was categorized as CKD if the albumin-creatinine ratio was ≥30 mg/g or the GFR was <60 ml/min per 1.73 m^2^ (Levey et al., 2009). ‘Weight in kilograms’ was divided by ‘height in meters squared’ to obtain body mass index (BMI). The BMI cutoffs to define under-/normal weight, overweight, and obesity were <25, 25–29.9, and ≥30 kg/m^2^, respectively. NHANES also reports family income to poverty ratio. This is the ratio of family's income and poverty threshold based on the number of family members; a higher ratio indicates a higher income (Ostchega et al., 2018; U.S. Department of Health and Human Services, 2019). This ratio was stratified as ‘<2’ and ‘≥2’. Participants were also asked about the number of health care visits they have done over the past year; which was categorized as 0, 1–3, and ≥4 (Ostchega et al., 2018). CVD event was defined if a person had a history of myocardial infarction, coronary heart disease, stroke, or heart failure. Among people without CVD event, 10-year predicted CVD risk was obtained with pooled cohort risk equations (Goff et al., 2014). Previous CVD event or ≥10% 10-year CVD risk was considered as high CVD risk (Muntner et al., 2017; Whelton et al., 2017).

Persons with stage 2 hypertension, stage 1 hypertension with diabetes, CKD, or high CVD risk, and SBP ≥130 mmHg with ≥65 years of age were considered as treatment-eligible for hypertension (Supplemental Table 1). Among individuals who reported that they were currently taking antihypertensive drugs, the following individuals were considered as not meeting treatment goals: if the SBP/DBP was ≥130/80 mmHg among age groups <65 years and if the SBP was ≥130 mmHg among age groups ≥65 years (Supplemental Table 1) (Muntner et al., 2017; Whelton et al., 2017).

### 2.3 Statistical analysis

The background characteristics of the hypertensive and overall study participants were reported according to their race. Mean and standard error (SE) were used to report continuous variables while categorical variables were reported with weighted percentages and unweighted numbers. During comparison, continuous variables were tested with *t*-tests and categorical variables were tested with chi-square tests. Then, the age-adjusted proportion (with 95% confidence interval [CI]) of individuals with hypertension, people with treatment-eligibility for hypertension, people who were not taking antihypertensive drugs despite being treatment-eligible, and people with unmet treatment goals among treated were reported according to race as well as the overall population. The age distributions from the 2015 population census were used to obtain age-adjusted estimates (US Census Bureau, 2015). The analysis also accounted for multistage cluster sampling design of the NHANES to obtain all the estimates (Johnson et al., 2014); using the mobile examination center's weights, all the weighted estimates were reported. Overall, due to a lower proportion of missing data (<10%), variables with missing data were neither dropped nor imputed (i.e., available case analysis) (Kang, 2013). Stata 14.0 was used to analyze data. The ‘svy’ command in Stata allows to adjust for the multistage cluster-sampling design (Stata Corporation, College Station, Texas USA, 2015).

## 3 Results

The analysis included 16,103 respondents aged ≥20 years (Table 1). The mean age of the participants was 47.6 years (SE: 0.4). About 51.8% of the respondents were females. Most of the characteristics differed according to race (*p* < 0.05). The proportion of hypertensive people with a high cholesterol level was higher among non-Hispanic whites while Mexican-Americans had a higher proportion of hypertensive people with young age, diabetes, obesity, and low family income to poverty ratio. Overall, 24.8% people had high CVD risk.

**Table 1:** Background characteristics of the hypertensive study participants by race, NHANES 2011–16^a^, ^b^.

Characteristics	Overall study population (*N* = 16,103)	Hypertensive participants by race					
		Non-Hispanic White (*n* = 3132/6066)	Non-Hispanic Blacks, (*n* = 2253/3665)	Mexican-Americans, (*n* = 981/2164)	Other races/ethnicities c, (*n* = 1885/4208)	All races, (*n* = 8251/16,103)	*p*-Value^d^
**SBP**, mean (SE), mmHg	122.4 (0.3)	133.0 (0.5)	136.8 (0.6)	134.8 (0.8)	134.8 (0.6)	133.9 (0.4)	<0.01
**DBP**, mean (SE), mmHg	70.7 (0.2)	74.4 (0.4)	76.1 (0.6)	76.1 (0.7)	76.2 (0.6)	74.9 (0.3)	<0.01

**Age (in years)**							
Mean (SE)	47.6 (0.4)	57.8 (0.4)	52.8 (0.6)	49.9 (1.1)	53.6 (0.7)	56.1 (0.3)	<0.001
20–44	44.7 (6797)	19.8 (587)	29.1 (493)	39.1 (229)	29.5 (414)	23.6 (1723)	<0.001
45–54	18.7 (2720)	18.9 (487)	23.8 (445)	23.3 (178)	20.7 (354)	20.1 (1464)	
55–64	17.3 (2798)	25.5 (593)	24.9 (634)	19.2 (271)	21.7 (480)	24.5 (1978)	
≥65	19.2 (3788)	35.8 (1465)	22.2 (681)	18.4 (303)	28.0 (637)	31.8 (3086)	

**Gender**							
Male	48.2 (7811)	51.0 (1616)	46.2 (1133)	56.5 (516)	52.6 (963)	50.9 (4228)	<0.001
Female	51.8 (8292)	49.0 (1516)	53.8 (1120)	43.5 (465)	47.4 (922)	49.1 (4023)	

**Cholesterol level (in mg/dl)**							
Normal (<200)	45.9 (7039)	29.7 (905)	43.5 (832)	37.8 (312)	34.8 (568)	32.6 (2617)	<0.001
Borderline (200–239)	24.0 (3504)	23.0 (631)	19.7 (398)	26.8 (229)	23.8 (423)	22.9 (1681)	
High (≥240)	30.0 (4734)	47.4 (1470)	36.8 (848)	35.5 (391)	41.4 (780)	44.5 (3489	

**High-density lipoprotein cholesterol (in mg/dl)**							
Normal	70.5 (10357)	67.9 (1924)	72.1 (1467)	60.9 (565)	62.8 (1096)	67.4 (5052)	<0.001
Low (<40 for men & <50 for women)	29.5 (4671)	32.1 (1003)	27.9 (526)	39.1 (356)	37.2 (624)	32.6 (2509)	

**Chronic kidney disease**							
No	85.1 (12981)	76.4 (2118)	75.5 (1583)	76.9 (706)	78.2 (1408)	76.5 (5815)	0.47
Yes	14.9 (2814)	23.6 (928)	24.5 (586)	23.1 (254)	21.8 (416)	23.5 (2184)	

**Diabetes mellitus status**							
No	83.4 (12515)	75.7 (2229)	71.1 (1466)	68.7 (596)	71.2 (1270)	74.0 (5561)	<0.001
Prediabetes	6.1 (950)	8.2 (238)	6.8 (144)	7.1 (70)	9.4 (160)	8.1 (612)	
Diabetes	10.5 (2221)	16.1 (550)	22.1 (548)	24.2 (284)	19.4 (387)	17.9 (1769)	

**Body mass index (in kg/m2)**							
Normal/underweight (<25)	29.5 (4695)	19.5 (642)	18.4 (421)	10.4 (108)	28.5 (552)	19.8 (1723)	<0.001
Overweight (25–29.9)	32.8 (5038)	34.1 (1000)	25.9 (590)	31.5 (311)	31.2 (615)	32.5 (2516)	
Obese (≥30)	37.8 (6013)	46.4 (1368)	55.7 (1164)	58.1 (533)	40.2 (649)	47.7 (3714)	

**Number of health care visits within the past year**							
0	15.3 (2600)	9.3 (276)	12.1 (246)	21.6 (168)	16.7 (282)	11.4 (972)	<0.001
1–3	64.8 (10264)	63.7 (1904)	67.5 (1528)	61.4 (608)	62.7 (1194)	63.9 (5234)	
≥4	19.9 (3223)	27.0 (948)	20.4 (478)	17.0 (203)	20.6 (407)	24.7 (2036)	

**Family income-to-poverty ratio**							
<2	36.7 (7305)	29.1 (1331)	53.7 (1083)	64.6 (566)	46.2 (821)	36.6 (3801)	0.07
≥2	63.3 (7383)	70.9 (1609)	46.3 (925)	35.4 (291)	53.8 (836)	63.4 (3661)	

**10-yr CVD risk categories**							
Low	75.2 (9652)	53.7 (1159)	55.8 (865)	70.1 (460)	60.4 (845)	55.9 (3329)	<0.001
High	24.8 (4322)	46.3 (1520)	44.2 (988)	29.9 (374)	39.3 (694)	44.1 (3576)	

Abbreviations: CVD: Cardiovascular disease, DBP: Diastolic blood pressure, NHANES: National Health and Nutrition Examination Survey, SBP: Systolic blood pressure, SE: Standard error.

a Numbers are presented as weighted percentages and unweighted numbers unless otherwise specified.

b Numbers may not add up to total because of missing values.

c Other races/ethnicities include non-Hispanic Asians, other Hispanics, and other races including multi-races.

d *p*-Values obtained by chi-square tests (for categorical variables) or t-tests (continuous variables).

**Fig. 1:**
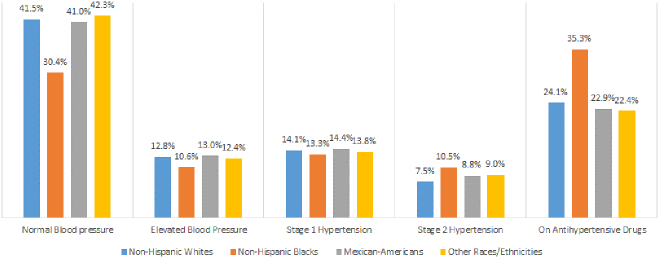
Age-adjusted prevalence of blood pressure stages according to race, National Health and Nutrition Examination Survey 2011–16.

Table 2 shows the age-adjusted prevalence of hypertension according to race and the prevalence among the overall population. The prevalence of hypertension was 45.7% (95% CI: 44.1%–47.3%) among non-Hispanic whites, 59.0% (95% CI: 57.4%–60.6%) among non-Hispanic blacks, 46.1% (95% CI: 43.8%–48.3%) among Mexican-Americans, 45.2% (95% CI: 43.3%–47.2%) among other races/ethnicities, and 46.9% (95% CI: 45.8%–48.1%) among overall population. In all race categories, males had a higher prevalence of hypertension compared to females. The characteristics (e.g., overweight/obesity, high cholesterol, CKD, or the number of health care visits) that had higher prevalence in one racial category also had higher prevalence in other racial categories. Regardless of characteristics, non-Hispanic blacks had the highest prevalence among all races. For some of the characteristics like high CVD risk, diabetes mellitus, CKD, and high cholesterol level, about three-fourths of non-Hispanic blacks had hypertension. Figure 1 shows the age-adjusted prevalence of blood pressure stages by race. The overall prevalence of stage 1 hypertension was similar across races; however, the prevalence of stage 2 hypertension and the proportion of people on antihypertensive medication was highest among non-Hispanic blacks.

**Table 2:** Age-adjusted prevalence (with 95% confidence interval) of hypertension according to race, NHANES 2011–16^a^.

Characteristics	Non-Hispanic Whites	Non-Hispanic Blacks	Mexican-Americans	Other races/ethnicities^b^	In all races
**Age (in years)**					
20–44	24.6 (22.3–27.0)	32.3 (29.5–35.2)	22.3 (19.5–25.4)	21.2 (18.6–24.0)	24.7 (23.0–26.4)
45–54	47.8 (43.3–52.4)	68.3 (63.9–72.4)	49.0 (43.2–54.7)	46.4 (42.6–50.3)	50.1 (47.3–53.0)
55–64	63.8 (60.4–67.1)	83.8 (80.5–86.7)	66.1 (60.1–71.5)	65.6 (61.0–70.0)	66.3 (63.8–68.8)
≥65	75.8 (73.1–78.4)	89.7 (87.3–91.8)	79.8 (73.1–85.2)	80.9 (77.3–84.1)	77.7 (75.3–79.8)

**Sex**					
Male	49.3 (46.9–51.7)	60.9 (58.9–62.9)	48.2 (45.3–51.2)	49.1 (46.6–51.7)	50.2 (48.4–51.9)
Female	42.0 (40.1–43.9)	57.4 (55.2–59.7)	43.6 (41.3–46.0)	41.6 (39.4–43.8)	43.7 (42.3–45.1)

**Cholesterol level (in mg/dl)**					
Normal (<200)	40.3 (37.9–42.8)	56.0 (53.9–58.2)	39.4 (37.0–42.0)	41.3 (38.2–44.5)	42.2 (40.4–44.1)
Borderline (200–239)	44.4 (41.4–47.4)	55.6 (52.1–59.1)	45.7 (40.0–51.6)	44.9 (41.2–48.6)	45.2 (42.9–47.5)
High (≥240)	57.9 (54.3–61.5)	73.5 (68.3–78.1)	59.1 (53.4–64.7)	56.2 (52.1–60.3)	59.2 (56.3–61.9)

**High-density lipoprotein cholesterol (in mg/dl)**					
Normal	42.0 (40.1–43.9)	56.6 (54.6–58.5)	44.8 (42.2–47.4)	43.0 (41.0–45.1)	43.8 (42.4–45.2)
Low (<40 for men & <50 for women)	53.8 (51.5–56.1)	64.9 (62.0–67.8)	47.1 (43.2–51.0)	49.4 (46.7–52.1)	53.2 (51.5–54.9)

**Chronic kidney disease**					
No	43.7 (42.0–45.4)	56.4 (54.6–58.3)	42.8 (40.9–44.8)	43.0 (40.9–45.1)	44.6 (43.3–45.9)
Yes	58.7 (53.8–63.4)	74.1 (69.2–78.5)	65.5 (60.4–70.3)	61.5 (56.4–66.3)	62.2 (58.9–65.4)

**Diabetes mellitus status**					
No	42.8 (40.9–44.6)	56.3 (54.1–58.4)	42.5 (40.0–44.9)	42.6 (40.4–44.8)	43.9 (42.6–45.3)
Prediabetes	54.1 (46.8–61.1)	64.8 (53.2–74.9)	52.0 (41.7–62.1)	57.2 (51.8–62.4)	55.8 (50.8–60.6)
Diabetes	73.9 (67.8–79.2)	76.9 (68.8–83.4)	65.8 (58.6–72.4)	57.5 (49.8–64.8)	70.2 (66.3–73.8)

**Body mass index (in kg/m^2^)**					
Normal/underweight (<25)	32.4 (30.0–34.8)	49.7 (46.8–52.6)	32.8 (28.0–38.1)	36.8 (33.7–40.0)	34.6 (32.8–36.5)
Overweight (25–29.9)	44.7 (42.4–46.9)	53.9 (51.7–56.1)	42.3 (39.0–45.6)	44.0 (41.8–46.1)	44.9 (43.3–46.5)
Obese (≥30)	57.9 (55.6–60.0)	67.3 (64.9–69.6)	53.1 (50.0–56.1)	57.1 (53.4–60.7)	58.2 (56.6–59.7)

**Family income to poverty ratio**					
<2	47.8 (45.3–50.4)	60.4 (58.2–62.7)	47.2 (44.7–49.7)	46.1 (43.9–48.3)	49.5 (48.1–50.9)
≥2	45.2 (43.2–47.2)	57.7 (55.3–60.0)	44.5 (41.1–47.9)	43.9 (41.0–47.0)	45.8 (44.3–47.3)

**Number of health care visits within the past year**					
0	42.6 (37.4–47.9)	58.5 (53.8–63.1)	.40.8 (36.4–45.3)	40.4 (35.3–45.8)	43.2 (39.8–46.7)
1–3	44.4 (42.7–46.2)	58.1 (56.5–59.7)	46.7 (44.0–49.5)	44.5 (42.0–47.0)	46.0 (44.6–47.4)
≥4	50.4 (46.9–53.9)	64.1 (60.1–67.9)	49.8 (44.2–55.4)	48.9 (44.5–53.3)	51.4 (48.7–54.1)

**10-yr CVD risk categories**					
Low	36.1 (33.9–38.3)	51.6 (48.3–54.8)	39.7 (36.9–42.6)	36.0 (33.5–38.7)	37.3 (35.6–39.0)
High	63.0 (56.3–69.2)	85.7 (78.9–90.6)	61.5 (49.3–72.5)	68.6 (59.2–76.7)	67.4 (62.7–71.8)
**Overall**	45.7 (44.1–47.3)	59.0 (57.4–60.6)	46.1 (43.8–48.3)	45.2 (43.3–47.2)	46.9 (45.8–48.1)

Abbreviations: CVD: Cardiovascular disease, NHANES: National Health and Nutrition Examination Survey.

a If the systolic/diastolic blood pressure cutoff was ≥130/80 mmHg or a person reported taking antihypertensive medication. This includes both stages 1 and 2 hypertension.

b Other races/ethnicities include non-Hispanic Asians, other Hispanics, and other races including multi-races.

The proportion of age-adjusted treatment-eligible individuals was 35.7% (95% CI: 34.3%–37.2%), 49.3% (95% CI: 47.7%–50.8%), 36.6% (95% CI: 34.1%–39.2%), 36.0% (95% CI: 34.3%–37.8%), and 37.2% (95% CI: 36.0%–38.3%) among non-Hispanic whites, non-Hispanic blacks, Mexican-Americans, other races/ethnicities, and the overall population, respectively (Table 3). Similar to estimates from Table 2, characteristics that had a higher prevalence of hypertension also had increased indication for treatment, and non-Hispanic blacks were more likely to be eligible compared to other races as per most characteristics.

**Table 3:** Age-adjusted proportion (with 95% confidence interval) of individuals require antihypertensive medication with characteristics stratified by race, NHANES 2011–16^a^.

Characteristics	Non-Hispanic Whites	Non-Hispanic Blacks	Mexican-Americans	Other races/ethnicities^b^	In all races
**Age (in years)**					
20-44	12.2 (10.5-14.0)	19.3 (17.1-21.6)	11.2 (9.3-13.5)	10.1 (8.6-11.8)	12.6 (11.4-13.9)
45-54	32.4 (28.6-36.4)	53.4 (48.9-57.9)	32.4 (26.3-39.3)	33.0 (29.5-36.8)	34.9 (32.3-37.7)
55-64	55.0 (51.0-59.0)	77.0 (73.0-80.5)	57.1 (50.6-63.3)	55.4 (50.7-60.0)	57.6 (54.6-60.5)
≥65	75.1 (72.4-77.6)	89.2 (86.5-91.5)	79.8 (73.1-85.2)	80.6 (76.8-83.8)	77.0 (74.7-79.2)

**Sex**					
Male	37.7 (35.7-39.8)	48.9 (46.9-50.8)	36.0 (32.6-39.5)	37.3 (35.1-39.5)	38.4 (36.9-40.1)
Female	33.6 (32.0-35.3)	49.5 (47.4-51.6)	37.1 (34.6-39.7)	34.9 (32.8-37.1)	35.8 (34.5-37.2)

**Cholesterol level (in mg/dl)**					
Normal (<200)	31.1 (28.9-33.5)	45.7 (43.0-48.3)	30.7 (28.0-33.6)	33.2 (30.5-36.0)	33.2 (31.5-35.0)
Borderline (200-239)	32.7 (29.8-35.7)	43.9 (41.3-46.5)	35.0 (28.9-41.5)	33.5 (30.5-36.7)	33.6 (31.4-35.8)
High (≥240)	48.5 (44.8-52.2)	64.3 (59.3-69.0)	47.3 (41.7-52.9)	45.9 (41.7-50.2)	49.3 (46.2-52.3)

**High-density lipoprotein cholesterol (in mg/dl)**					
Normal	32.6 (30.7-34.6)	46.5 (44.5-48.6)	35.3 (32.6-38.1)	33.4 (31.6-35.3)	34.3 (32.8-35.8)
Low (<40 for men & <50 for women)	43.0 (40.4-45.6)	55.8 (52.6-59.0)	38.8 (35.6-42.1)	41.4 (38.4-44.5)	43.5 (41.6-45.4)

**Chronic kidney disease**					
No	32.7 (31.3-34.2)	45.2 (43.3-47.0)	32.2 (30.0-34.5)	32.8 (31.0-34.8)	33.8 (32.6-35.0)
Yes	58.7 (53.8-63.4)	74.1 (69.2-78.5)	65.5 (60.4-70.3)	61.5 (56.4-66.3)	62.2 (58.9-65.4)

**Diabetes mellitus status**					
No	31.7 (30.1-33.3)	45.0 (43.1-47.0)	31.6 (28.9-34.3)	32.4 (30.3-34.6)	33.0 (31.7-34.2)
Prediabetes	54.1 (46.8-61.1)	64.8 (53.2-74.9)	52.0 (41.7-62.1)	57.2 (51.8-62.4)	55.8 (50.8-60.6)
Diabetes	68.0 (60.7-74.5)	72.5 (64.7-79.0)	55.8 (49.2-62.3)	51.5 (43.7-59.2)	63.9 (59.5-68.0)

**Body mass index (in kg/m**^**2**^**)**					
Normal/underweight (<25)	24.5 (22.5-26.7)	40.3 (37.7-43.0)	25.4 (20.9-30.6)	28.7 (26.1-31.5)	26.6 (25.0-28.2)
Overweight (25-29.9)	33.4 (31.9-35.0)	43.4 (41.0-45.8)	32.8 (29.2-36.6)	35.0 (32.7-37.4)	34.3 (33.1-35.6)
Obese (≥30)	46.2 (44.2-48.1)	57.5 (55.2-59.7)	42.9 (39.7-46.1)	45.9 (42.7-49.2)	47.1 (45.6-48.7)

**Family income to poverty ratio**					
<2	39.6 (37.5-41.8)	51.1 (49.1-53.0)	37.0 (34.0-40.0)	37.8 (35.4-40.2)	40.8 (39.4-42.2)
≥2	34.4 (32.6-36.2)	47.8 (45.6-50.0)	35.7 (33.0-38.6)	34.2 (31.6-36.8)	35.3 (33.9-36.8)

**Number of health care visits within the past year**					
0	29.1 (25.2-33.4)	42.3 (39.1-45.8)	30.6 (25.7-35.9)	27.7 (22.5-33.6)	30.1 (27.1-33.2)
1-3	34.6 (32.9-36.3)	49.1 (47.4-50.8)	36.6 (33.9-39.3)	35.9 (33.7-38.2)	.36.5 (35.1-37.9)
≥4	42.5 (39.4-45.7)	58.2 (54.0-62.3)	45.6 (39.9-51.5)	42.8 (38.9-46.7)	44.2 (41.7-46.7)

**10-yr CVD risk categories**					
Low	23.7 (21.7-25.8)	37.6 (33.5-41.9)	28.5 (25.1-32.2)	24.8 (22.1-27.8)	25.0 (23.3-26.7)
High	63.0 (56.3-69.2)	85.7 (78.9-90.6)	61.5 (49.3-72.5)	68.6 (59.2-76.7)	67.4 (62.7-71.8)
**Overall**	35.7 (34.3-37.2)	49.3 (47.7-50.8)	36.6 (34.1-39.2)	36.0 (34.3-37.8)	37.2 (36.0-38.3)

Abbreviations: CVD: Cardiovascular disease, NHANES: National Health and Nutrition Examination Survey.

a Individuals with stage 2 hypertension, stage 1 hypertension with diabetes, chronic kidney disease or high CVD risk, and systolic blood pressure ≥ 130 mmHg with ≥65 years of age were considered as treatment-eligible for hypertension.

b Other races/ethnicities include non-Hispanic Asians, other Hispanics, and other races including multi-races.

Table 4 shows the age-adjusted proportion of individuals who were not receiving treatment despite having indication; contrary to the findings of Table 2, Table 3, a lower proportion of non-Hispanic blacks were untreated regardless of characteristics. Overall, about half of the people with other races/ethnicities were not receiving treatment, followed by Mexican-Americans, non-Hispanic whites, and non-Hispanic blacks, 49.3% (95% CI: 45.5%–53.0%), 46.5% (95% CI: 42.0%–51.0%), 38.9% (95% CI: 35.3%–42.6%), and 35.1% (95% CI: 32.4%–37.9%), respectively. In all races, younger people, males, people with normal cholesterol, normal HDL, non-diabetic, normal weight, low family income to poverty ratio, and low CVD risk were more likely to remain untreated despite having treatment-indication. For most of the characteristics, despite having indications for treatment, Mexican-Americans and people of other races/ethnicities had a greater proportion of untreated people compared to the other 2 races.

**Table 4:** Age-adjusted proportion (with 95% confidence interval) of untreated hypertensive adults among who have antihypertensive treatment indication per characteristics stratified by race, NHANES 2011–16^a^.

Characteristics	Non-Hispanic Whites	Non-Hispanic Blacks	Mexican-Americans	Other races/ethnicities^b^	In all races
**Age (in years)**					
20-44	48.6 (42.1-55.1)	47.9 (41.9-53.9)	58.1 (48.7-67.0)	65.7 (58.1-72.6)	51.9 (47.6-56.2)
45-54	37.2 (31.2-43.7)	28.3 (22.0-35.6)	50.0 (39.8-60.2)	44.2 (36.5-52.2)	37.5 (33.4-41.7)
55-64	29.1 (24.9-33.6)	24.2 (19.7-29.3)	30.2 (22.8-38.8)	34.1 (28.2-40.5)	29.0 (25.9-32.2)
≥65	27.0 (23.6-30.7)	22.1 (19.2-25.2)	30.8 (26.5-35.6)	29.8 (26.0-33.8)	27.0 (24.3-29.8)

**Sex**					
Male	45.9 (41.5-50.2)	42.9 (38.0-48.0)	56.4 (49.1-63.5)	52.9 (47.9-57.7)	47.3 (44.4-50.3)
Female	29.5 (25.0-34.4)	28.7 (24.9-32.8)	34.8 (27.2-43.2)	45.4 (39.4-51.5)	32.3 (29.2-35.5)

**Cholesterol level (in mg/dl)**					
Normal (<200)	41.4 (36.5-46.5)	37.8 (33.7-42.0)	47.4 (39.1-55.8)	56.8 (50.3-63.0)	43.2 (39.9-46.6)
Borderline (200-239)	49.4 (41.2-57.7)	49.2 (42.0-56.4)	55.5 (43.9-66.6)	56.9 (49.9-63.6)	50.8 (45.3-56.3)
High (≥240)	28.2 (24.2-32.7)	17.8 (13.0-24.0)	39.1 (28.8-50.5)	40.1 (30.4-50.5)	29.4 (26.0-33.0)

**High-density lipoprotein cholesterol (in mg/dl)**					
Normal	41.3 (36.7-46.0)	37.2 (33.6-40.9)	51.9 (44.0-59.8)	52.7 (47.8-57.7)	43.0 (39.9-46.1)
Low (<40 for men & <50 for women)	34.2 (28.7-40.2)	28.3 (23.6-33.5)	37.8 (30.8-45.4)	44.4 (37.9-51.1)	35.4 (31.6-39.4)

**Chronic kidney disease**					
No	36.3 (32.3-40.6)	33.8 (30.6-37.2)	43.6 (37.8-49.7)	48.4 (44.2-52.7)	38.3 (35.4-41.2)
Yes	53.7 (46.9-60.3)	40.3 (35.9-44.8)	51.5 (42.7-60.1)	52.9 (43.9-61.6)	50.2 (46.5-54.0)

**Diabetes mellitus status**					
No	41.9 (38.2-45.7)	40.7 (38.0-43.5)	53.7 (46.7-60.6)	57.2 (52.9-61.4)	44.6 (42.3-47.0)
Prediabetes	40.3 (30.9-50.5)	38.8 (28.6-50.1)	53.3 (38.2-67.8)	42.4 (30.8-54.9)	41.1 (34.7-47.9)
Diabetes	25.1 (16.0-37.2)	17.5 (11.4-25.9)	28.5 (19.5-39.8)	27.2 (14.5-45.2)	24.2 (18.6-30.8)

**Body mass index (in kg/m**^**2**^**)**					
Normal/underweight (<25)	47.4 (36.7-58.3)	55.9 (47.1-64.4)	61.4 (40.5-78.8)	58.8 (52.2-65.1)	51.2 (43.8-58.5)
Overweight (25-29.9)	42.0 (34.6-49.7)	31.4 (24.3-39.5)	59.0 (46.5-70.4)	54.4 (47.6-61.0)	43.9 (39.0-49.0)
Obese (≥30)	34.9 (31.0-39.1)	31.6 (28.2-35.2)	39.4 (33.0-46.2)	42.7 (36.4-49.2)	35.7 (33.1-38.3)

**Family income to poverty ratio**					
<2	41.3 (36.6-46.1)	35.9 (30.6-41.6)	48.9 (40.3-57.6)	51.6 (47.1-56.1)	42.3 (39.4-45.3)
≥2	38.0 (33.3-42.9)	34.7 (30.5-39.2)	43.4 (33.8-53.5)	48.5 (42.0-54.9)	39.2 (35.7-42.8)

**Number of health care visits within the past year**					
0	82.1 (72.8-88.7)	85.3 (75.4-91.6)	92.4 (84.5-96.4)	87.2 (77.4-93.1)	87.2 (77.4-93.1)
1-3	38.1 (33.3-43.1)	32.6 (29.3-36.0)	39.7 (32.0-47.9)	47.0 (42.3-51.9)	47.0 (42.3-51.9)
≥4	25.2 (20.2-30.8)	21.3 (16.1-27.7)	25.0 (14.6-39.2)	33.9 (26.3-42.5)	33.9 (26.3- 42.5)

**10-yr CVD risk categories**					
Low	41.1 (35.4-47.0)	34.8 (30.6-39.2)	48.3 (41.1-55.5)	52.1 (46.4-57.8)	42.6 (38.9-46.4)
High	36.7 (26.2-48.7)	25.2 (17.3-35.1)	43.8 (28.1-60.8)	52.2 (43.0-61.3)	37.6 (31.6-43.9)
**Overall**	38.9 (35.3-42.6)	35.1 (32.4-37.9)	46.5 (42.0-51.0)	49.3 (45.5-53.0)	40.4 (38.0-42.8)

Abbreviations: CVD: Cardiovascular disease, NHANES: National Health and Nutrition Examination Survey.

a Individuals with stage 2 hypertension, stage 1 hypertension with diabetes, chronic kidney disease or high CVD risk, and systolic blood pressure ≥ 130 mmHg with ≥65 years of age were considered as treatment-eligible for hypertension.

b Other races/ethnicities include non-Hispanic Asians, other Hispanics, and other races including multi-races.

**Fig. 2:**
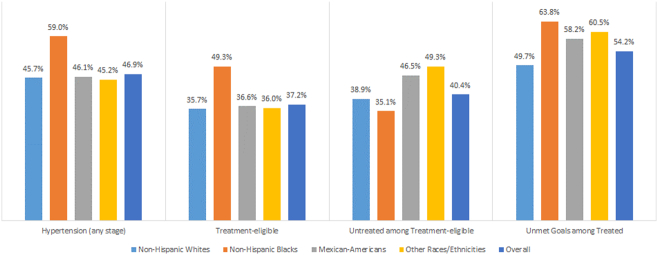
Age-adjusted proportion of people with hypertension, treatment-eligible for hypertension, untreated among treatment-eligible, and unmet treatment goals among treated according to race, National Health and Nutrition Examination Survey 2011–16.

As shown in Table 5, according to race, ordered from the highest age-adjusted proportion, 63.8% (95% CI: 60.0%–67.5%) of non-Hispanic blacks, 60.5% (95% CI: 54.1%–66.5%) of other races/ethnicities, 58.2% (95% CI: 51.7%–64.5%) of Mexican-Americans, and 49.7% (95% CI: 45.0%–54.4%) of non-Hispanic whites were not meeting the treatment goals. Per most of the characteristics, non-Hispanic blacks had higher proportions of people with unmet treatment goals compared to other 3 races. Figure 2 summarizes all four studied prevalence according to race as well as the overall population.

**Table 5:** Age-stratified and overall proportion (with 95% confidence interval) of treated hypertensive adults who have unmet treatment goals per characteristics stratified by race, NHANES 2011–16^a^.

Characteristics	Non-Hispanic Whites	Non-Hispanic Blacks	Mexican-Americans	Other races/ethnicities^b^	In all races
**Age (in years)**					
20-44	45.9 (36.2-55.9)	61.6 (54.8-67.9)	60.2 (42.5-75.6)	62.2 (49.1-73.7)	52.2 (45.4-58.9)
45-54	46.8 (37.6-56.2)	64.9 (58.7-70.6)	47.9 (30.4-66.0)	56.4 (47.7-64.8)	51.7 (45.6-57.8)
55-64	48.6 (42.2-55.1)	62.0 (57.4-66.5)	57.1 (49.5-64.3)	53.8 (44.2-63.2)	51.6 (46.9-56.4)
≥65	61.7 (57.3-66.0)	69.5 (65.5-73.2)	64.2 (54.1-73.3)	66.0 (59.8-71.6)	63.1 (59.4-66.5)

**Sex**					
Male	52.8 (45.6-59.8)	68.7 (62.3-74.4)	66.5 (53.2-77.6)	61.8 (52.9-70.0)	57.1 (52.0-62.1)
Female	46.7 (41.5-51.9)	61.0 (55.2-66.5)	53.3 (44.1-62.3)	59.0 (49.8-67.7)	51.8 (48.0-55.7)

**Cholesterol level (in mg/dl)**					
Normal (<200)	50.3 (42.0-58.6)	63.0 (55.8-69.7)	62.1 (50.4-72.5)	64.4 (54.1-73.4)	55.3 (49.5-61.0)
Borderline (200-239)	50.2 (40.2-60.1)	75.1 (61.1-85.3)	51.6 (38.0-65.0)	64.2 (50.3-76.0)	55.6 (48.6-62.4)
High (≥240)	48.0 (40.8-55.1)	61.0 (52.7-68.8)	57.0 (32.1-78.8)	56.4 (42.0-69.9)	51.4 (45.7-57.1)

**High-density lipoprotein cholesterol (in mg/dl)**					
Normal	47.9 (42.9-53.0)	64.8 (58.7-70.5)	59.0 (46.8-70.2)	60.6 (49.6-70.7)	55.4 (50.8-59.8)
Low (<40 for men & <50 for women)	63.6 (47.9-76.9)	61.1 (52.8-68.9)	55.8 (46.6-64.6)	58.5 (49.1-67.3)	51.4 (46.6-56.3)

**Chronic kidney disease**					
No	48.7 (43.6-53.8)	61.8 (56.4-66.9)	57.6 (50.6-64.3)	58.2 (51.3-64.8)	52.7 (48.8-56.5)
Yes	56.9 (42.7-70.1)	67.0 (57.0-75.6)	59.2 (36.6-78.6)	68.0 (53.3-79.8)	60.3 (53.0-67.1)

**Diabetes mellitus status**					
No	49.1 (43.6-54.6)	62.3 (56.7-67.6)	61.3 (51.5-70.2)	65.9 (58.3-72.7)	53.8 (49.6-58.0)
Prediabetes	49.1 (34.5-63.8)	64.0 (43.9-80.1)	71.6 (44.6-88.8)	55.3 (40.1-69.6)	54.3 (45.1-63.1)
Diabetes	52.3 (42.0-62.3)	68.0 (60.4-74.7)	50.0 (31.9-68.2)	51.8 (36.3-66.9)	55.1 (48.1-62.0)

**Body mass index (in kg/m**^**2**^**)**					
Normal/underweight (<25)	47.1 (34.6-60.0)	64.6 (46.8-79.1)	72.2 (63.0-79.8)	57.1 (41.1-71.7)	52.3 (42.1-62.4)
Overweight (25-29.9)	49.3 (41.0-57.7)	69.6 (60.7-77.3)	50.1 (33.9-66.3)	66.7 (53.3-77.9)	54.6 (47.8-61.3)
Obese (≥30)	50.2 (44.7-55.7)	62.1 (57.5-66.4)	57.2 (49.0-65.0)	57.0 (50.0-63.7)	54.1 (50.4-57.6)

**Family income to poverty ratio**					
<2	46.1 (39.2-53.1)	64.3 (57.9-70.3)	56.9 (46.5-66.8)	58.2 (49.2-66.6)	54.4 (50.0-58.8)
≥2	50.3 (44.3-56.3)	64.8 (59.8-69.5)	56.9 (42.8-69.9)	63.1 (53.3-72.0)	53.7 (49.1-58.1)

**Number of health care visits within the past year**					
0	57.4 (30.8-80.4)	84.5 (69.5-92.8)	30.4 (20.5-42.4)	87.5 (57.8-97.3)	60.2 (42.0-76.0)
1-3	49.5 (43.5-55.6)	64.5 (59.7-69.1)	61.6 (51.6-70.7)	58.0 (51.0-64.7)	54.8 (50.4-59.1)
≥4	49.2 (41.6-56.8)	60.0 (53.5-66.1)	54.7 (38.7-69.8)	64.2 (53.0-74.1)	52.4 (46.9-57.9)

**10-yr CVD risk categories**					
Low	40.4 (33.4-47.7)	45.5 (40.2-51.0)	45.0 (29.7-61.3)	51.6 (43.2-59.9)	43.1 (37.8-48.5)
High	51.5 (41.6-61.3)	76.3 (65.6-84.5)	65.0 (39.7-84.0)	68.2 (50.7-81.8)	60.7 (53.9-67.1)
**Overall**	49.7 (45.0-54.4)	63.8 (60.0-67.5)	58.2 (51.7-64.5)	60.5 (54.1-66.5)	54.2 (50.7-57.6)

Abbreviations: CVD: Cardiovascular disease, NHANES: National Health and Nutrition Examination Survey.

a If the systolic/diastolic blood pressure was ≥130/80 mmHg among individuals taking any BP lowering drugs (age groups <65 years) or the systolic blood pressure was ≥130 mmHg (age groups ≥65 years), then they were considered as persons with unmet treatment goals.

b Other races/ethnicities include non-Hispanic Asians, other Hispanics, and other races including multi-races.

## 4 Discussion

This study revealed the racial disparities in age-adjusted prevalence and treatment status of hypertension within the US. Compared to other races, non-Hispanic blacks had higher prevalence and treatment indication for hypertension. Although they were more likely to be treated, about two-thirds were not meeting treatment goals; this proportion was also the highest among all races. The proportion of individuals who could be hypertensive or treatment-eligible for hypertension among races other than the non-Hispanic blacks were similar; however, the proportion of untreated individuals despite being treatment-eligible was substantially higher among Mexican-Americans and other races/ethnicities. To the best of this author's knowledge, this is the first epidemiological study to report the racial/ethnic disparities in prevalence, treatment, and control of hypertension among US adults per the 2017 ACC/AHA guideline.

The overall estimates of prevalence and treatment status observed by this study are similar to findings of other studies (Dorans et al., 2018; Khera et al., 2018; Muntner et al., 2017). Over the past few decades, non-Hispanic blacks had the highest prevalence and unmet treatment goals among all races in the US (Dorans et al., 2018; Whelton et al., 2017). However, the prevalence of hypertension among Africans in Africa are considerably lower than this prevalence. Although genetic similarities exist between Africans in Africa and African-Americans in the US, differences in lifestyles and socioeconomic factors between the African-Americans and Africans in Africa could cause this higher prevalence of hypertension (Dorans et al., 2018; Forouzanfar et al., 2017; Ostchega et al., 2018; Whelton et al., 2017). These findings highlight the importance of adopting adequate lifestyle and other preventive measures that could reduce the higher prevalence of hypertension among African-Americans.

The prevalence of overall and uncontrolled hypertension among Mexican-Americans was higher than the prevalence estimates by Muntner and colleagues that also analyzed the NHANES datasets from 2011 to 14 survey years (Muntner et al., 2017); this could be due to age-adjustment. Without age-adjustment, a large proportion of younger people among Mexican-Americans could show a lower prevalence of hypertension among them (Table 1). Dorans et al. reported the age-adjusted prevalence of hypertension according to race following application of the 2017 guideline. The estimates reported by the authors were similar to the estimates of this study (Dorans et al., 2018).

Even though the non-Hispanic blacks were receiving treatment, the treatment was not adequate to reduce the pressure level (i.e., to meet the treatment goals). Regular monitoring of treated individuals is thus essential. This proportion was also substantially higher among people from other races/ethnicities as well as Mexican-Americans. The uncontrolled hypertension could put them at a greater risk of complications as also seen by previous studies (Muntner et al., 2017; Ostchega et al., 2018). Even a large proportion of people with high CVD risks had uncontrolled hypertension. Furthermore, the overall age-adjusted proportion of untreated treatment-eligible people was more than 40%, this proportion was higher among other races/ethnicities and Mexican-Americans; the age-adjusted number of health care visits over the past year was also lower among them (Supplemental Table 2). A previous study by Gu et al. suggests that a large proportion of uninsured participants among these races could be responsible for this underutilization of treatment (Gu et al., 2017). Multiple individual, social, and healthcare-related factors could also cause this underutilization (Egan et al., 2014; Gu et al., 2017; Wang and Vasan, 2005). Ensuring adequate treatment for all these untreated and uncontrolled individuals by overcoming these barriers would be helpful to reduce the complications of hypertension including the disparities associated with treatment.

Despite the dissimilarities, all four age-adjusted proportions were substantially higher among all studied races. To prevent and treat hypertension or its complications, all hypertensive individuals need to adopt lifestyle and other preventive measures such as increasing physical activity or restricting salt intake (Whelton et al., 2017). Additionally, controlling other associated conditions such as diabetes, dyslipidemia, increased body weight, or CKD that increases likelihoods of hypertension would be essential. Some of the conditions such as CVD risk and CKD were disproportionately higher among non-Hispanic blacks compared to other 3 races (Supplemental Table 2), that could cause the difference. Preventing and treating these conditions would be necessary to reduce the racial disparities associated with prevention, treatment, and control of hypertension and its complications including the overall burden. These conditions also share common risk factors; the prevention and control strategies of these conditions are similar to hypertension (Berrington de Gonzalez et al., 2010; Burke et al., 2008; Forouzanfar et al., 2015; Hypertension Study Group, 2001; Scholes et al., 2012).

If any of the participants of the present study (i.e., NHANES 2011–16) ever received an evaluation, were evaluated or treated under previous guidelines such as the JNC 7 guideline (Chalmers et al., 1999; Chobanian et al., 2003). As those guidelines recommend different cutoffs to define hypertension, treatment indication, and treatment goals, reevaluation of previously treated people is thus important. Despite a substantial increase in prevalence of hypertension following application of the 2017 ACC/AHA guideline, the overall proportion of treatment-eligible people would be similar to previous guidelines (Muntner et al., 2017). Furthermore, people who would be classified as stage 1 hypertensive per the 2017 ACC/AHA guideline, were classified as prehypertensive per the previous guidelines (Whelton et al., 2017). Previous guidelines also recommend preventive measures for prehypertensive individuals (Chalmers et al., 1999; Chobanian et al., 2003). However, adoption of the new guideline could substantially reduce the complications resulting from hypertension and increase the awareness among all people from early stages of hypertension (Bundy et al., 2018; Whelton et al., 2017; Yano et al., 2018). Thus, the new guideline has potentials to reduce the racial disparities associated with hypertension and its complications if it is implemented, and adequate preventive and treatment measures are taken (Muntner et al., 2017; Whelton et al., 2017).

Racial disparities in prevalence, treatment, and control of hypertension and other chronic diseases have been studied widely over the past two decades (Giles et al., 2007; Gu et al., 2017; Karlamangla et al., 2010; Redmond et al., 2011). Studies have also shown how the awareness or outcome of diseases differ (Batson et al., 2010; Giles et al., 2007; Gu et al., 2017). Interventions are needed to uptake preventive and treatment measures as well as awareness. Prevention and control programs should incorporate the findings of the present study to reduce these disparities. Strategies such as prescribing (e.g., once-daily regimen if possible), educating (e.g., clearly written instructions), and tracking/encouraging (e.g., encouraging patients to use reminders) have been shown effective to increase medication adherence (Chang et al., 2018). These strategies require interactions of patients, physicians, and health care delivery systems (Bradbury et al., 2018; Chang et al., 2018). Successful implementation of these strategies is essential to reduce the racial disparities associated with prevention and treatment measures including the overall burden. In addition to races that have poor treatment or control of hypertension, people with the characteristics that were more likely to remain untreated (e.g., younger age and male gender) or uncontrolled (e.g., CKD and high CVD risk) are essential to prioritize.

This study has several notable strengths. First, the estimates were derived from three large nationally representative samples. This survey (i.e., NHANES) used standardized validated methods to obtain BP levels. Using adequate statistical procedures to adjust for sample weights and age along with a large sample size also enabled reporting of prevalence on a wide range of background characteristics (Johnson et al., 2014; Muntner et al., 2017). The limitations of the present study also merit discussion. The NHANES data was cross-sectional, and the BP was measured on a single day; this could cause some overestimation as the 2017 ACC/AHA recommends using measures of multiple days to confirm the diagnosis of hypertension (Muntner et al., 2017; Whelton et al., 2017). Moreover, as earlier studies found a higher proportion of untreated people among Hispanics other than Mexican-Americans, grouping them with non-Hispanic Asians and other races as a separate category (i.e., other races/ethnicities) could lead to some overestimation in that group.

## 5 Conclusion

This study reported the racial/ethnic disparities associated with prevalence, treatment, and control of hypertension among US adults per the 2017 ACC/AHA guideline. Although the age-adjusted prevalence of hypertension was substantially higher among all races, the non-Hispanic blacks had the highest prevalence regardless of characteristics. Treatment and control disparities also exist where the majority of non-Hispanic blacks were not meeting the treatment goals, and a substantial proportion of Mexican-Americans or people of ‘other races/ethnicities’ were not receiving treatment despite being eligible for antihypertensive treatment. Interventions are needed to increase prevention, treatment, and control measures by all races to reduce racial disparities associated with hypertension burden, including the complications resulting from it.

The following are the supplementary data related to this article.

Supplemental Table 1 Definition of outcome variables. Supplemental Table 1

Supplemental Table 2 Age-stratified proportion of adults with selected characteristics stratified by race, NHANES 2011–16. Supplemental Table 2

## Author's contributions

GMAK had full access to all the data in the study and takes responsibility for the integrity of the data and the accuracy of the data analysis.

Concept, first draft, statistical analysis, writing, review, and editing: GMAK

## Conflicts of interest

No conflicts of interest to disclose.

## Funding

Not received for this study.
