# Supplementary material for: Racial/ethnic disparities in prevalence, treatment, and control of hypertension among US adults following application of the 2017 American College of Cardiology/American Heart Association guideline
Source: Prev Med Rep. 2019 Mar 16;14:100850. doi: 10.1016/j.pmedr.2019.100850 (PMC6488531; doi:10.1016/j.pmedr.2019.100850)
Supplement: Supplemental Table 1 — Definition of outcome variables. [file mmc1.docx]

| Supplemental Table 1: Definition of outcome variables | |
| --- | --- |
| Variable Name | Definition |
| Elevated BP | Systolic BP within the range of 120-129 mm Hg with a Diastolic BP of <80 mm Hg. |
| Stage 1 hypertension | The systolic/diastolic BP is 130-139/80-89 mm Hg. |
| Stage 2 hypertension | The systolic/diastolic BP is above ≥140/90 mm Hg. |
| Hypertension (any type) | Stage 1 or stage 2 hypertension or the participant reported taking antihypertensive agents. |
| Treatment eligible for hypertension | Individuals with stage 2 hypertension; stage 1 hypertension with diabetes, chronic kidney disease or ≥10% 10 year cardiovascular disease risk; and SBP ≥130 mm Hg with 65 years or older people. |
| Persons with unmet treatment goals | Among individuals currently reported taking any BP lowering drugs, if the systolic/diastolic BP was ≥130/80 mm Hg (among individuals below 65 years) or if the systolic BP was ≥130 mm Hg (among individuals 65 years or older). |
