# Supplementary material for: Racial/ethnic disparities in prevalence, treatment, and control of hypertension among US adults following application of the 2017 American College of Cardiology/American Heart Association guideline
Source: Prev Med Rep. 2019 Mar 16;14:100850. doi: 10.1016/j.pmedr.2019.100850 (PMC6488531; doi:10.1016/j.pmedr.2019.100850)
Supplement: Supplemental Table 2 — Age-stratified proportion of adults with selected characteristics stratified by race, NHANES 2011–16. [file mmc2.docx]

| **Supplemental Table 2: Age-stratified proportion of adults with selected characteristics stratified by race, NHANES 2011-16** | | | | | |
| --- | --- | --- | --- | --- | --- |
|  | **Non-Hispanic Whites** | **Non-Hispanic Blacks** | **Mexican-Americans** | **Other races/ethnicities** | **p-values^1^** |
| **Cholesterol level (in mg/dl)** | | |  |  |  |
| Normal (<200) | 45.0% | 52.1% | 47.8% | 46.3% | <0.001 |
| Borderline elevated (200-239) | 24.3% | 20.6% | 24.6% | 23.9% |  |
| High (≥240) | 30.7% | 27.4% | 27.6% | 29.8% |  |
| **High-density lipoprotein cholesterol (in mg/dl)** | | | | |  |
| Normal | 71.5% | 74.7% | 63.2% | 66.5% | <0.001 |
| Low (<40 for men and <50 for women) | 28.5% | 25.3% | 36.8% | 33.5% |  |
| **Chronic kidney disease** | | |  |  |  |
| No | 85.7% | 81.4% | 83.6% | 85.5% | <0.001 |
| Yes | 14.3% | 18.7% | 16.4% | 14.5% |  |
| **Diabetes mellitus status** | | | |  |  |
| No | 85.2% | 78.0% | 75.6% | 79.7% | <0.001 |
| Prediabetes | 5.9% | 5.7% | 6.2% | 6.9% |  |
| Diabetes | 8.9% | 16.3% | 18.2% | 13.5% |  |
| **Body mass index (in kg/m^2^)** |  |  |  |  |  |
| Normal/underweight (<25) | 30.4% | 24.3% | 17.2% | 38.1% | <0.001 |
| Overweight (25-29.9) | 33.5% | 28.2% | 35.0% | 31.6% |  |
| Obese (≥30) | 36.2% | 47.5% | 47.8% | 30.3% |  |
| **Family income to poverty ratio** | |  |  |  |  |
| <2 | 29.2% | 54.2% | 64.0% | 45.2% | <0.001 |
| 2 or more | 70.8% | 45.8% | 36.0% | 54.8% |  |
| **Number of health care visits within the past year** | | | | | |
| 0 | 12.9% | 13.9% | 24.5% | 19.6% | <0.001 |
| 1-3 | 65.3% | 68.4% | 59.5% | 63.3% |  |
| ≥4 | 21.8% | 17.7% | 16.0% | 17.1% |  |
| **10-yr CVD-risk categories** | | | |  |  |
| Low | 75.2% | 67.3% | 73.5% | 74.0% | <0.001 |
| High | 24.8% | 32.7% | 26.5% | 26.0% |  |

Abbreviations: CVD: Cardiovascular disease; NHANES: National Health and Nutrition Examination Survey

1. P-values obtained by chi-square tests
